# Occult pulmonary mucosa-associated lymphoid tissue lymphoma presenting as catastrophic antiphospholipid antibody syndrome

**DOI:** 10.3892/ol.2013.1585

**Published:** 2013-09-13

**Authors:** HARIHARAN REGUNATH, JAMES SHORTRIDGE, SHAHZAD RAZA, PUJA NISTALA, BRANDON M. HUFFMAN, MICHAEL X. WANG, DONG XIANG

**Affiliations:** 1 Department of Internal Medicine, University of Missouri, Columbia, MO 65203, USA; 2 Division of Hematology and Medical Oncology, Ellis Fischel Cancer Center, University of Missouri, Columbia, MO 65203, USA; 3 Department of Pathology and Anatomical Sciences, Ellis Fischel Cancer Center, University of Missouri, Columbia, MO 65203, USA

**Keywords:** lymphoma, antiphospholipid antibody, rituximab, plasmapheresis

## Abstract

Catastrophic antiphospholipid antibody syndrome (CAPS) is characterized by fulminant thrombosis of the arterial and venous beds of multiple organ systems over a relatively short period of time and with a high mortality rate. Mucosa-associated lymphoid tissue (MALT) lymphoma of the lung has never been reported as a causative or precipitating factor for CAPS in the CAPS registry database. The present study describes a rare case of pulmonary MALT lymphoma of the lung that presented as CAPS. A 19-year-old Hispanic female presented with shortness of breath and abdominal pain. Computed tomography (CT) scans of the chest and abdomen revealed multiple portal vein thromboses and bilateral pulmonary nodules. Within one week of presentation, the patient developed a straight sinus thrombosis and upper extremity deep vein thrombosis, which led to shortness of breath. A biopsy of the lung nodule revealed MALT lymphoma. The present case illustrates a rarely reported pulmonary MALT lymphoma presenting as CAPS in a young female. The patient was successfully treated with 90 mg/m^2^ bendamustine on days one and two and rituximab 375 mg/m^2^ on day one of each 28-day cycle. Complete remission of the lung nodules was observed following three cycles of treatment, as visualized by positron emission tomography (PET)/CT scan. Fondaparinux was identified as a feasible anticoagulation drug of choice for this case. At seven months post-treatment, the patient continues to be stable with no further evidence of thrombosis and is currently undergoing rituximab maintenance therapy every six months for two years. A repeat lupus anticoagulant antibody assay turned and remained negative during the clinical follow-up period. A prompt diagnosis and early aggressive treatment is potentially curative and may dramatically decrease the mortality risk. Future studies should explore the role of rituximab in the management of CAPS-associated B-cell lymphoid malignancies.

## Introduction

Catastrophic antiphospholipid antibody syndrome (CAPS) is a life-threatening variant of antiphospholipid antibody syndrome (APLA) (1). The condition is typically characterized by fulminant thrombosis of the arterial and venous beds of multiple organ systems over a relatively short period of time. Although it has been reported to occur in a small percentage of patients with APLA syndrome, the cognizance of this condition is vital, as early treatment with anticoagulation therapies, aggressive immunosuppression or plasmapheresis may decrease morbidity and mortality rates (1,2). The present study describes a case of mucosa-associated lymphoid tissue (MALT) lymphoma of the lung that presented as CAPS and was successfully treated using a combination of plasmapheresis, rituximab and fondaparinux anticoagulation, leading to a resolution of a life-threatening event. Written informed consent was obtained from the patient.

## Case report

**Figure 1:**
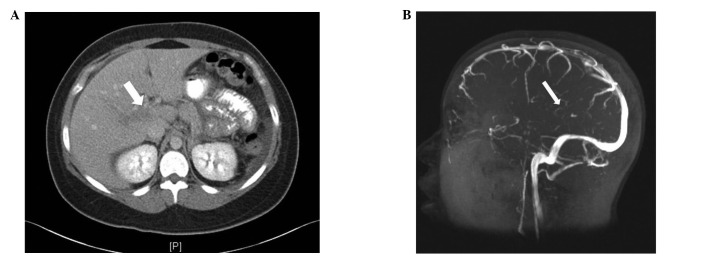
(A) Contrast abdominal CT scan showing a hypodensity and the absence of intravenous contrast noted in the area of the portal vein (white arrow). (B) MRV showing the absence of significant flow in the straight sinus, indicating a thrombosis (white arrow). CT, computed tomography; MRV, magnetic resonance venography.

A 19-year-old Hispanic female with a past history of Evan’s syndrome was referred to the University of Missouri Hospital (Columbia, MO, USA) with abdominal pain associated with fever, nausea, vomiting, coughing and hematochezia. A diagnosis of a portal vein thrombosis was confirmed using an abdominal computed tomography (CT) scan (Fig. 1A) and duplex ultrasonogram one day prior to the presentation to the hospital. On admission, the patient was dyspneic with 96% SPO_2_ in 2 l of oxygen. A full dose of enoxaparin was administered. A contrast chest CT was negative for pulmonary emboli, but disclosed multiple bilateral lung nodules that were distributed peripherally. The nodules measured 1.9×1.8 cm in the right upper lobe, 2.1×0.9 cm in the left upper lobe and 1.2×1.1 cm in the right lower lobe. A small amount of bowel thickening was noted on the external CT scan. The upper and lower endoscopies were unremarkable, with the exception of the presence of gastropathy. The immunological work-up was positive for the lupus anticoagulant.

On day four, the patient experienced blurred vision in the left eye due to bilateral papilledema, as revealed by an ophthalmoscopic exam. Brain magnetic resonance imaging (MRI) showed a T2/fluid-attenuated inversion recovery (FLAIR) high-signal abnormality involving the left temporal lobe. Within the next 24 h, the symptoms of abdominal pain, hematochezia and headache worsened. The brain MRI was repeated using magnetic resonance venography (MRV), which revealed a thrombosis of the straight sinus (Fig. 1B) with a probable involvement of the veins of Rosenthal, which drain the temporal lobes. A sub-acute venous infarct in the left temporal lobe was also observed. A contrast CT scan of the abdomen revealed a new colonic wall thickening with a notable extension of the portal vein thrombus into the superior mesenteric vein and further caudally, placing the patient at a high risk for ischemic bowel necrosis. Due to the risk of bowel gangrene, following the correction of the international normalized ratio (INR), an exploratory laparotomy was attempted, revealing 40 cm of necrotic ischemic bowel with no evidence of vasculitis. CAPS was suspected based on the development of venous thromboses in the brain, portal vein and intestine, with a positive lupus anticoagulant assay in a short time-span. The patient was initially administered i.v. heparin and plasmapheresis without steroids due to the concern over wound healing issues.

On the sixth post-operative day, the patient experienced severe hematemesis and hematochezia, therefore, i.v. heparin was stopped. Emergent endoscopies did not reveal a definite source of the bleeding. A repeat abdominal CT scan showed the presence of an ileus and a small bowel lesion, suggesting a developing hematoma. However, the patient was managed conservatively and the condition improved. Four days later, deep vein thromboses developed in the left axillary, brachial and basilic veins. Heparin was restarted using a modified heparin procedure with a lower target partial thromboplastin time (PTT; 40–60 sec) compared with the usual institutional PTT range (60–85 sec). The range was tolerated without further bleeding or thrombosis. The PTT goal was then gradually increased to a range of 60–85 sec when the patient had been clinically stable for 48 h on the lower target range.

**Figure 2:**
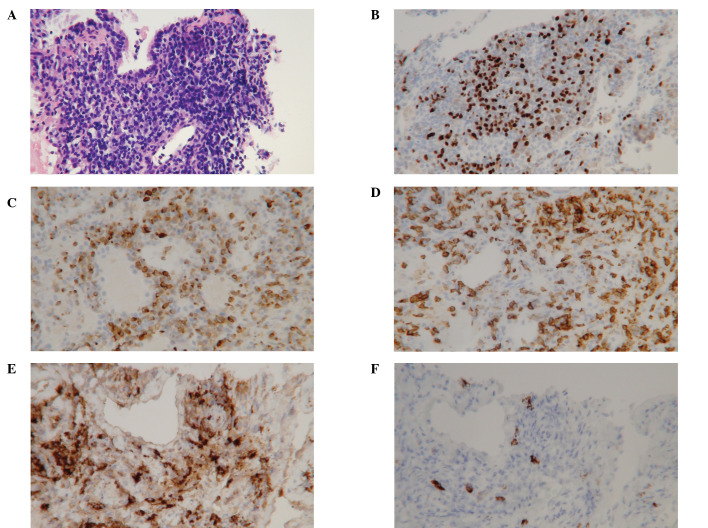
(A) Lymphoepithelial lesion with disruption of the bronchial epithelium and vessel by monocytoid lymphoma cells (H&E staining; original magnification, ×50). (B) PAX5 immunostain highlights small B-cell lymphocytes infiltrating the epithelium (original magnification, ×50). (C) Lymphoma cells were BCL2-positive and infiltrated the bronchial epithelium (IHC; original magnification, ×50). (D) Lymphoma cells with aberrant expression of CD43 in the lymphoepithelial lesion (IHC; original magnification, ×50). (E). Monoclonality of lymphoma cells is demonstrated by κ-light chain immunostain. In-situ hybridization study confirmed monotypic expression of κ immunoglobulin light chains. (IHC; original magnification, ×50). (F) Sparse immunostain of λ immunoglobulin light chain (IHC; original magnification, ×50). IHC, immunohistochemistry; H&E, hematoxylin and eosin.

To treat the suspected CAPS, the patient was administered a non-steroid immunosuppressant monoclonal antibody, rituximab, to avoid poor post-operative wound healing issues from steroid use. The patient continued to improve and was therefore discharged following three weeks of treatment. The patient underwent a percutaneous biopsy of a persistent right lower lobe lung nodule one month after being discharged. Histopathologically, the sections revealed lung parenchyma with bronchial epithelium and vessels that were heavily infiltrated by monocytoid small lymphocytes (Fig 2A). The lymphoid cells were positive for PAX5 (Fig. 2B), BCL2 (Fig. 2C), CD43 (Fig. 2D) and κ-light chain (Fig. 2E) immunostaining, and were negative for λ-light chain (Fig. 2F) immunostaining. The findings were consistent with a diagnosis of extranodal marginal zone lymphoma of respiratory MALT lymphoma.

The patient was treated with bendamustine at 90 mg/m^2^ on days one and two and 375 mg/m^2^ rituximab on day one of every 28-day cycle. Complete remission of the lung nodules was observed following three cycles of treatment, as visualized by positron emission tomography (PET)/CT scans. The anticoagulation regime was switched from enoxaparin to fondaparinux. The patient continues to be stable with no further evidence of thrombosis following seven months of treatment and is currently on rituximab maintenance therapy every six months for two years. A repeat lupus anticoagulant antibody assay turned and remained negative in the clinical follow-up appointments.

## Discussion

CAPS is a rare but serious complication of APLA that carries a significantly high mortality rate (50%) (2,4). According to the CAPS registry database, the disease is three times more common in females and is usually seen in patients aged ~40 years old (2). In cases from the CAPS registry, a precipitating factor was noted in 53% of cases with infections (22%), with recent surgery (10%) being the most common. Malignancy accounted for 9% of cases. Hematological malignancies were observed to be the most common (26%), followed by lung (17%) and colon (9%) cancer (2). A pulmonary MALT lymphoma has never been reported as a causative or precipitating factor for CAPS from this registry.

Although a clearly proposed criteria for the diagnosis of non-catastrophic APLA and CAPS exists, the two conditions represent opposite ends of the disease spectrum (5,6). The majority of cases in the CAPS registry were considered ‘probable CAPS’ primarily due to the lack of a tissue biopsy, which was avoided due to the requirement to stop anticoagulation treatment. The patient in the present study developed a thrombosis of the portal vein, dural venous sinuses and upper extremity deep veins. For the same reason as previously mentioned, a tissue biopsy was not obtained from the liver or lung to confirm the small vessel thrombosis that were shown with clear evidence on imaging studies.

Traditionally, plasmapheresis has been used for CAPS therapy (7,8). In recent years, the addition of rituximab to anticoagulants, steroids and plasmapheresis, has resulted in a reduction of the lupus anticoagulant levels back to normal. In non-catastrophic primary and secondary APS, rituximab therapy has improved the outcome. Four published cases have had a history of B-cell non-Hodgkin lymphoma (NHL), with two being large B-cell lymphomas and two being splenic marginal zone lymphomas (9,10).

Managing anticoagulation itself is very challenging in the event of CAPS. In the situation of an acute bleed that requires a transfusion, it is difficult to continue anticoagulation treatment and therefore, the patient faces the risk of thrombosis. In the present study, when anticoagulation treatment was stopped following the episode of hematemesis, the patient developed an axillary vein thrombosis within 24 hours. Compared with the institutional range of 60–85 sec, the lower target PTT (40–60 sec) strategy was well-tolerated without further bleeding or thrombosis. Such individualization of anticoagulation strategies may be required for managing CAPS where the coagulation system is in labile balance.

The present case is unique in that the age of presentation was younger than usual for malignancy-associated CAPS and also that CAPS was the presenting symptom, unlike those cases from the registry and the available studies where all cases had a proven prior history of cancer. CAPS should be suspected in any patient with clinical or radiological evidence of rapidly developing thromboses in multiple organs. The decision to treat the condition using aggressive measures should not be delayed until all criteria are met.

A thrombotic event that is associated with non-gastric MALT lymphoma may be the first manifestation of CAPS. A prompt diagnosis and early aggressive treatment is potentially curative and may dramatically decrease the mortality risk. Rituximab may be an effective adjuvant treatment for managing primary pulmonary MALT lymphoma when combined with bendamustine and fondaparinux. Future studies should explore the role of rituximab in the management of CAPS-associated B*-*cell lymphoid malignancies.
